# Electrochemical Detection of Solution Phase Hybridization Related to Single Nucleotide Mutation by Carbon Nanofibers Enriched Electrodes

**DOI:** 10.3390/ma12203377

**Published:** 2019-10-16

**Authors:** Arzum Erdem, Ece Eksin

**Affiliations:** 1 Faculty of Pharmacy, Analytical Chemistry Department, Ege University, Bornova, Izmir 35100, Turkey; 2 Biotechnology Department, Graduate School of Natural and Applied Sciences, Ege University, Bornova, Izmir 35100, Turkey; eceksin@hotmail.com

**Keywords:** carbon nanofibers, solution-phase nucleic acid hybridization, zip nucleic acids, screen printed electrodes, electrochemical impedance spectroscopy

## Abstract

In the present study, a sensitive and selective impedimetric detection of solution-phase nucleic acid hybridization related to Factor V Leiden (FV Leiden) mutation was performed by carbon nanofibers (CNF) modified screen printed electrodes (SPE). The microscopic and electrochemical characterization of CNF-SPEs was explored in comparison to the unmodified electrodes. Since the FV Leiden mutation is a widespread inherited risk factor predisposing to venous thromboembolism, this study herein aimed to perform the impedimetric detection of FV Leiden mutation by a zip nucleic acid (ZNA) probe-based assay in combination with CNF-SPEs. The selectivity of the assay was then examined against the mutation-free DNA sequences as well as the synthetic PCR samples.

## 1. Introduction

Nucleic acid-based technologies have been greatly used in research and diagnostics. In order to get the specific, accurate and sensitive analysis of nucleic acid hybridization, the specific sequence recognition is required to be improved. The efficiency of nucleic acid hybridization can be improved by decreasing the electrostatic repulsion between negatively charged nucleic acid strands. In this aspect, the coupling of oligonucleotides with spermine residues as the cationic units provider has been recently introduced in the literature [1,2,3].

Zip nucleic acids (ZNAs) contain a cationic compound, spermine. Thus, the affinity between ZNA probe and its target sequence increases by means of spermine. The melting temperature of oligonucleotide increases linearly with the number of conjugated spermine. Accordingly, a decrease at the electrostatic repulsion between the probe and its target sequence can be obtained [4]. They exhibit a high affinity to its target sequence while displaying a quite selective behavior to the single-base mutations [4].

For over a decade, scientists have focused on developing new technologies for single nucleotide polymorphisms (SNPs) analysis, such as, differential hybridization strategies, single-base extension after amplification, mismatch endonuclease-based detection and DNA sequencing with PCR, ligase chain reaction, and nick translation PCR with fluorogenic DNA probes [5,6,7]. In general, these conventional methods are labor-intensive, time-consuming and inefficient for diagnostics. For these reasons, there is urgent need to develop a novel specific and sensitive assay for real-time SNP detection.

Electrochemical biosensors provide a fast response with cost-effective measurement and less demanding instrumentation for mostly direct measurement of various analytes. According to the changes at the charge transfer resistance (R_ct_), the biorecognition process in biosensor applications could be analyzed as reported in earlier reports [8,9,10,11,12,13]. Under this aim, a meaningful difference between the R_ct_ obtained before and after nucleic acid (NA) hybridization is considered as an indicative response of full match hybridization [14]. In relation to the level of increase at the R_ct_ value that occurred as a result of double-stranded nucleic acids in comparison to single stranded probe, NA hybridization as well as SNP detection could be detected impedimetrically [14,15,16,17,18].

FV Leiden is a specific gene mutation as G-to-A substitution at nucleotide 1691 that predicts a single-amino acid replacement (R506Q) which results in thrombophilia [19,20]. The quantitative analysis of FV Leiden was performed by different techniques, such as, an immunosorbent assay [21], a fluorescent assay [22], the sandwich-optical sensing method [23] or a voltammetric assay [24,25]. Along with these conventional techniques, the impedance spectroscopy has an inherent potential for label-free detection, which is of special interest in bioanalysis since it circumvents the need to modify biomolecules with fluorescence dyes, enzymes, redox or radioactive labels [14]. The elimination of the label simplifies and speeds up the whole process and reduces the cost for each analysis.

According to one recent report [3], ZNA primers can improve the yield of cDNA synthesis for the genes that expressed at low concentration levels. Paris et al. [26] compared the performance of ZNA probes and minor groove binder probes. It was reported that ZNA probes can present a higher performance under standard PCR conditions. Alvandi et al. [27] performed real-time PCR using a ZNA probe and consequently, they verified that ZNA is reliable to be used as a real-time PCR probe. Accordingly, the study of Alvandi et al. [27] is further evidence for the accuracy of using ZNA to obtain hybridization with a high efficiency.

Carbon nanofibers (CNFs) are cylindrical nanostructures with graphene sheets that have lengths in the order of µm, as their diameter differs from 10 to 100 nm. Carbon nanofibers (CNFs) are significant nanomaterials for the design of novel (bio)sensor surfaces, because they provide high surface areas, non-toxicity, and acceptable biocompatibility [28,29]. Hence, CNFs with their excellent conductivity and structural properties provide a specific surface area, especially for nucleic acid biosensors and aptasensors [30,31,32,33].

To date, there has been no report introduced in the literature related to the impedimetric detection of solution-phase nucleic acid hybridization performed by ZNA in order to detect SNPs. In the case of solid-phase hybridization, a diffusion barrier significantly reduces the hybridization rate [34]. Thus, kinetically solution-phase hybridization is more preferentially used than the solid-phase hybridization. The inefficient hybridization with poor sensitivity is primarily due to the reason of steric hindrance and surface electrostatic forces affecting the ability of the target nucleic acids to their capturing probes [35]. Therefore, the solution-phase hybridization method was followed in our study to perform hybridization more efficiently before performing impedimetric detection. This is in contrast to our earlier reports presenting solid-phase hybridization in combination with voltammetric and impedimetric detection [36,37] and solution-phase hybridization in combination with voltammetric detection [38].

Under this aim, a ZNA probe was used herein for the development of an assay based on solution-phase nucleic acid hybridization related to single nucleotide mutation of FV Leiden. Before and after hybridization of the ZNA probe with an mDNA target specific to FV Leiden mutation, an impedimetric measurement was performed by using disposable electrodes, carbon nanofiber-screen printed electrodes (CNF-SPEs). The optimization of experimental conditions was performed in order to improve the sensitivity and selectivity of the impedimetric detection of hybridization. The selectivity of this assay was then examined against mutation-free DNA sequences as well as synthetic PCR samples.

## 2. Materials and Methods

### 2.1. Apparatus and Chemicals

The impedimetric measurements were performed by AUTOLAB-30 and AUTOLAB-302 PGSTAT with NOVA (version 1.1.2 EcoChemie, Utrecht, The Netherlands) and GPES 4.9.007 software package (EcoChemie, Utrecht, The Netherlands), respectively. All measurements were done in a Faraday cage (EcoChemie, Utrecht, The Netherlands).

The ZNA probe, lyophilized DNA oligonucleotides and PCR products were purchased from TIB Molbiol (Berlin, Germany). The details about the base sequences of oligonucleotides and PCR products are given in the Supporting Information.

The detailed information about CNF-SPEs is available in the Supporting Information.

### 2.2. Procedure

An impedimetric detection of NA includes the following steps,

(i) Hybridization of the ZNA probe with the mDNA target, or wDNA, non-complementary oligonucleotides (C-DNA, T-DNA, NC-1, NC-2), mutant type PCR products; mPCR-1 and mPCR-2 and wild type PCR products; wPCR-1 and wPCR-2 in the solution phase.
(ii) Immobilization of the hybrid of ZNA:DNA as well as others onto the surface of CNF-SPEs.
(iii) Measurements via electrochemical impedance spectroscopy (EIS) technique.

**Scheme 1:**
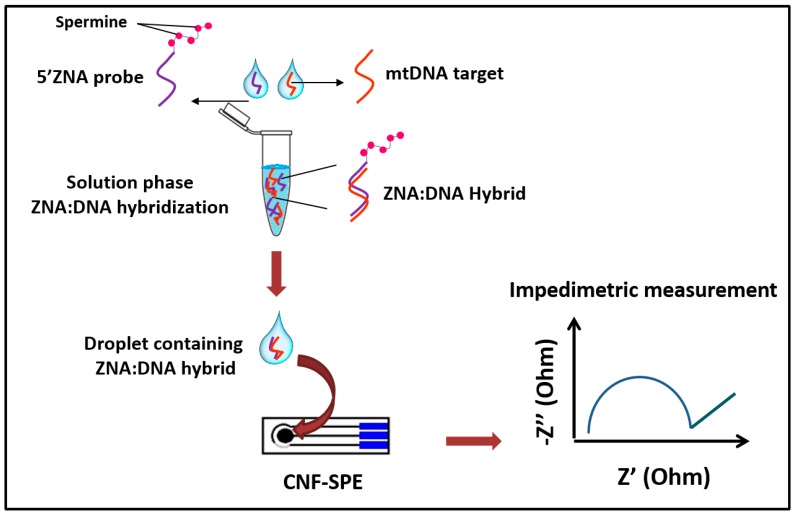
The schematic representation of the hybridization between 5’ zip nucleic acid (ZNA) probe and mDNA target in the solution phase and the immobilization of the hybrid onto the surface of carbon nanofiber-screen printed electrode (CNF-SPE) and the impedimetric measurement for the detection of the FV Leiden mutation.

The illustrative presentation of the impedimetric detection on the FV-Leiden mutation by CNF-SPEs is represented in Scheme 1.

The required amount of the ZNA probe (or DNA probe) and mDNA (or any other of DNA oligonucleotides) was mixed and allowed for solution-phase NA hybridization during 10 min. After then, the immobilization of the sample containing hybrids onto CNF-SPE was performed. The same experimental procedure was followed by using the DNA probe instead of the ZNA probe. Besides, the control experiments were performed by spermine alone. The sample containing the hybrid of ZNA:DNA (or DNA:DNA) was dropped onto the surface of a working electrode and kept during 15 min. Then, the electrodes were washed with buffer.

### 2.3. Impedimetric Measurements

The EIS measurements were performed according to the procedure reported in our previous work [37]. The impedance was measured in the frequency range between 100 kHz and 0.1 Hz. The Randles circuit was used as the equivalent circuit model used for fitting of the impedance data and comprised of the solution resistance (R_s_), the capacitance (Q), the charge transfer resistance (R_ct_) and the Warburg impedance (W). The respective semicircle diameter corresponds to R_ct_. The circuit model was used for fitting the impedance data as shown in all figures containing Nyquist diagrams.

## 3. Results and Discussion

**Figure 1:**
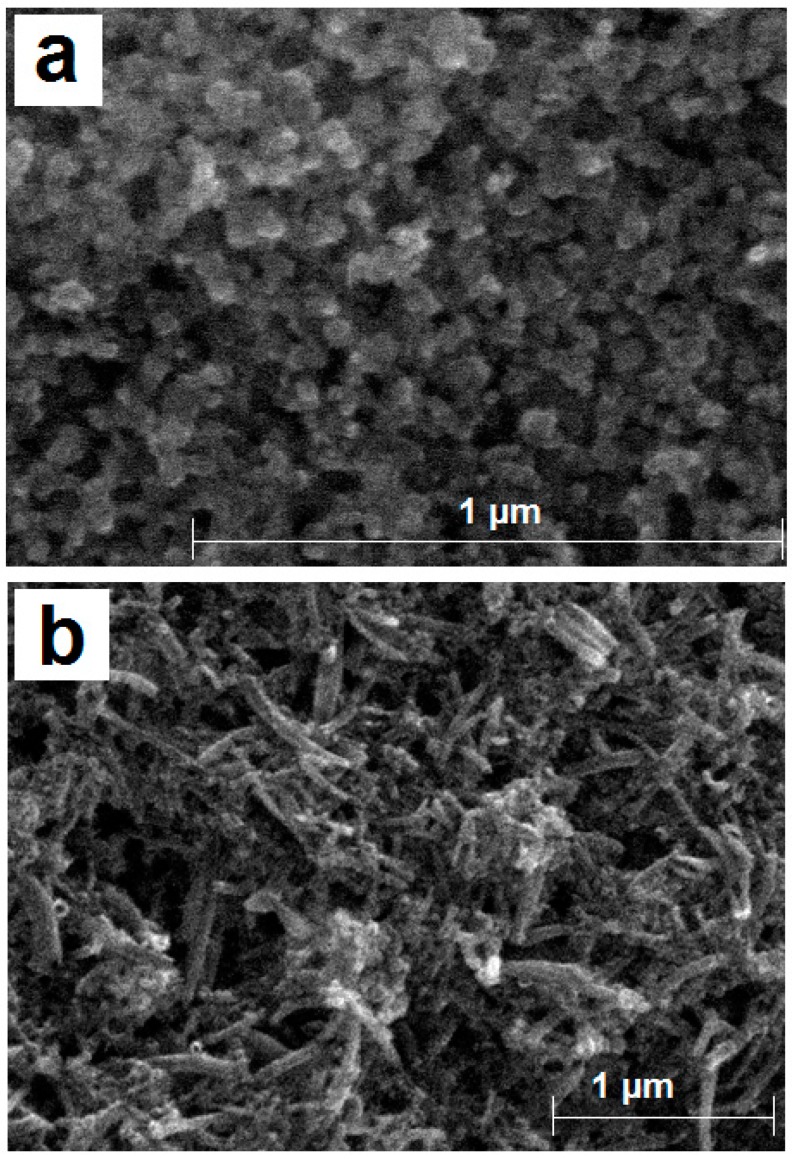
SEM images of (**a**) SPE using acceleration voltage as 5 kV, (**b**) CNF-SPE using acceleration voltage as 10 kV with the resolution of 1 µm.

The nanofiber structures of CNF-SPE at the resolution of 1 µm are clearly seen in Figure 1b in comparison to the SPE (shown in Figure 1a) according to the microscopic characterization of the electrodes by using SEM.

**Figure 2:**
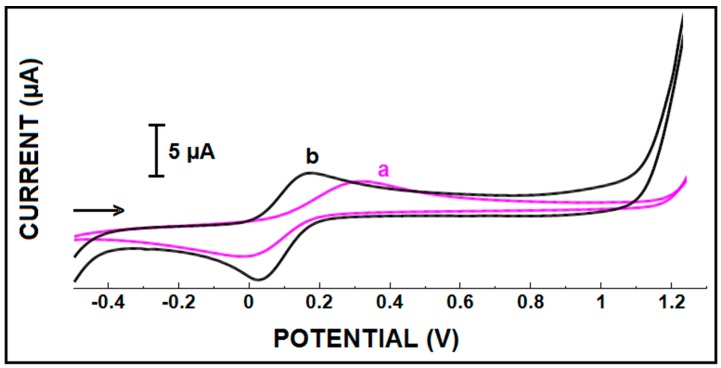
Cyclic voltammograms (CVs) of (**a**) SPE, (**b**) CNF-SPE in 2 mM K_4_[Fe(CN)_6_]/K_3_[Fe(CN)_6_] prepared in 0.1 M KCl.

The voltammetric measurements of SPE and CNF-SPE were performed via the cyclic voltammetry (CV) technique for the investigation of their electrochemical behavior (Figure 2). The anodic current I_a_, cathodic current I_c_ and the surface areas of the SPE and CNF-SPE are listed in Table 1. The anodic and cathodic currents that recorded by CNF-SPE were larger than the ones recorded by SPEs. Additionally, the electroactive surface areas (A) of SPE and CNF-SPE were calculated according to the Randles-Sevcik equation [39] and found to be 0.118 cm^2^ and 0.132 cm^2^, respectively. These results confirmed that the role of CNF is to accelerate the electron transfer and increase the surface area of the sensor which is similar to earlier reports [30,40,41,42,43,44,45].

**Figure 3:**
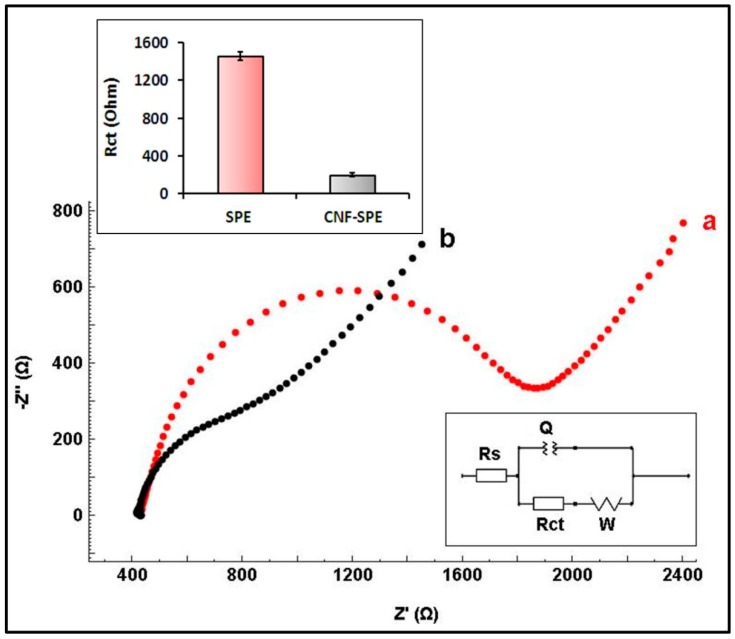
Nyquist diagrams of (**a**) SPE, (**b**) CNF-SPE. Inset: Histograms representing the average R_ct_ values recorded by (**a**) SPE, (**b**) CNF-SPE.

The EIS technique is used for electrochemical characterization of CNF-SPEs. The average R_ct_ value was obtained as 1455 ± 45.25 Ohm (RSD%, 3.11%, *n* = 3) by SPEs (Figure 3a). This is higher than the one measured by CNF-SPEs (i.e., 1198.50 ± 17.78 Ohm (RSD%, 8.96%, *n* = 3)) (Figure 3b). This decrease is a result of an enhanced electron transfer occurring between CNF-SPE and the electrolyte interface due to the conductive structure of CNFs [41,46,47,48].

In order to perform the selective and sensitive detection of solution-phase hybridization of ZNA:DNA, the experimental conditions (hybridization temperature, Mg^2+^ concentration, pH, hybridization time and probe concentration) were optimized. The results are represented in Supporting Information as Figures S1–S6.

**Figure 4:**
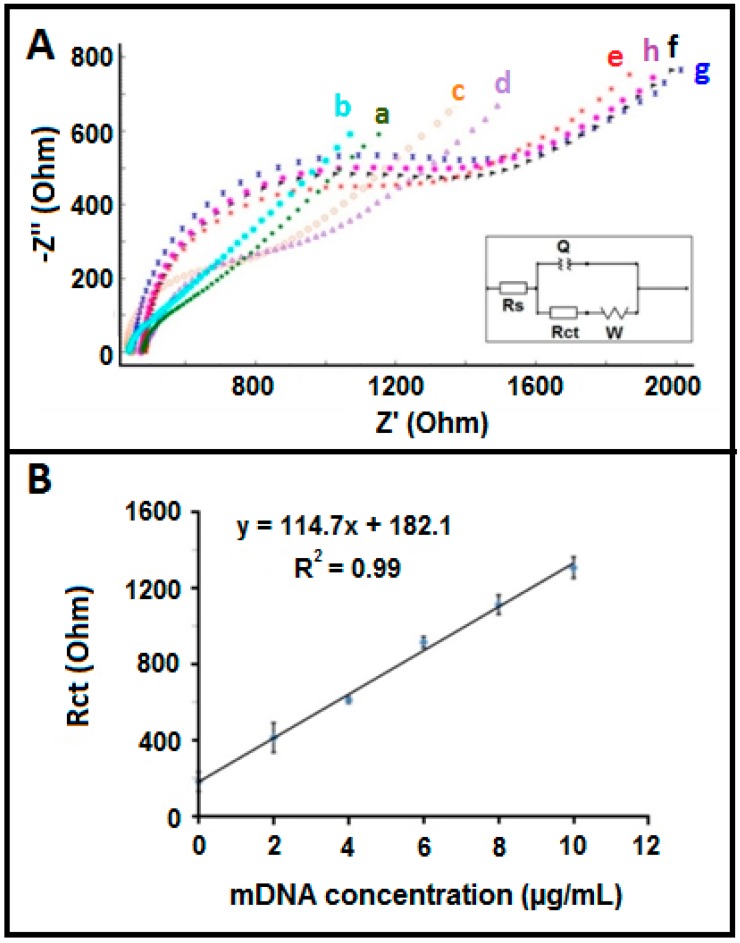
(**A**) Nyquist diagrams obtained by (a) CNF-SPE, (b) after the pseudo hybridization of ZNA probe, after the hybridization between ZNA probe and mDNA target in the concentration level of (c) 2, (d) 4, (e) 6, (f) 8, (g) 10, (h) 12 µg/mL. (**B**) Calibration graph based on the average R_ct_ values (*n* = 3) obtained after the hybridization of 1 µg/mL ZNA probe with mDNA target in the concentration range from 2 to 10 µg/mL.

The electrode surface was exposed to the hybrid of ZNA-DNA which occurred in the presence of different concentrations of the target DNA, and accordingly the change of R_ct_ value was recorded (Figure 4 and Figure S7). The hybridization between 1 µg/mL of the ZNA probe and the mDNA target in its different concentrations varying from 2 to 14 µg/mL (equals to 0.28 µM and 1.96 µM, respectively) was performed. The results are shown in Figure 4. There was a gradual increase obtained at R_ct_ from 2 to 10 µg/mL, and then a decrease at response was recorded at 12 µg/mL mDNA target (Figure S7). Since the highest R_ct_ value was obtained with the hybridization of 10 µg/mL mDNA target of all, 10 µg/mL (equals to 1.4 µM) mDNA target was chosen herein for our further studies.

The intra-day reproducibility of the results measured by CNF-SPEs during three days was calculated based on R_ct_ values obtained in the case of a full-match hybridization of the probe with the mDNA target by CNF-SPEs (shown in Table S1). The RSD % values varied from 3.08 % to 6.73 %. In order to examine the inter-day reproducibility, these results were combined and accordingly, the average R_ct_ value was calculated and found to be 1277.83 ± 62.54 Ohm with the RSD % of 4.89 % (*n* = 6) (shown in Table S2). The results revealed that the CNF-SPE’s exhibited a satisfactory reproducibility with a mean change of the response as 62.55 Ohm and a relative standard deviation of 4.89 %.

According to data presented in the calibration graph (Figure 4B), the respective linear regression equation expressed as y = 114.7x + 182.1; R^2^ = 0.99. The limit of detection (LOD) of the sensor was calculated was calculated according to the Miller and Miller method [49] and found to be 0.69 µg/mL (i.e., 96.5 nM, 1.93 pmol in 20 µL sample). Thus, the CNF-SPE possesses good electrocatalytic parameters for the detection of mDNA in terms of a wide linear range with a low LOD.

**Figure 5:**
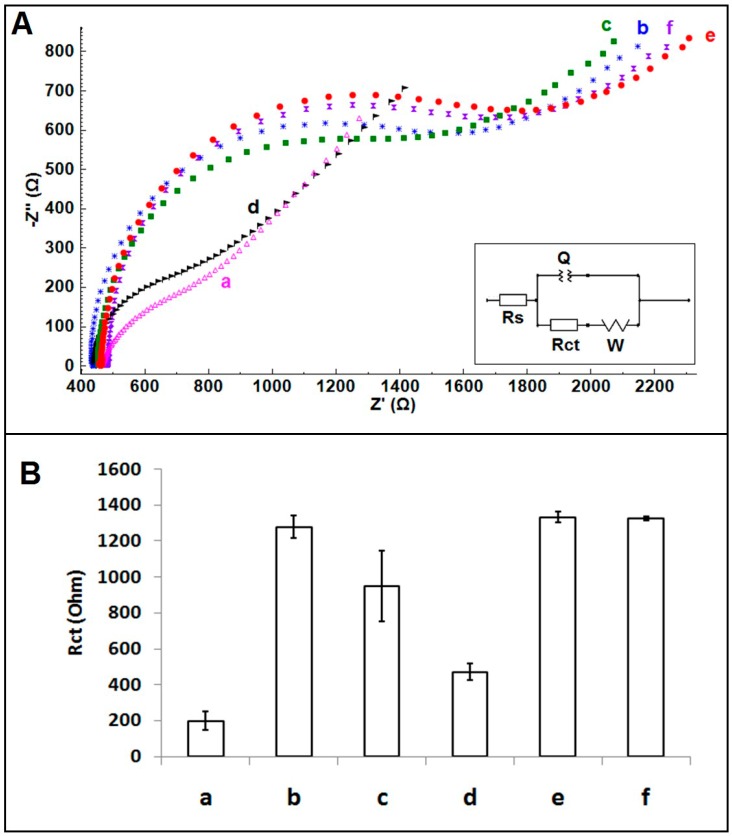
(**A**) Nyquist diagrams, (**B**) Histograms representing pseudo hybridization of (a) 1 µg/mL ZNA probe, after hybridization of 1 µg/mL ZNA probe with 10 µg/mL (b) mDNA target, (c) wDNA target, after pseudo hybridization of (d) 1 µg/mL DNA probe, after hybridization of 1 µg/mL DNA probe with 10 µg/mL (e) mDNA target, (f) wDNA target.

The selectivity of solution phase-nucleic acid hybridization related to single nucleotide mutation was tested in the presence of wild type DNA target. In addition, the same experimental procedure was also followed in the presence of the DNA probe in contrast to the ZNA probe. After hybridization of the ZNA probe with the mDNA target (Figure 5), there was a 5.4 fold increase at R_ct_ recorded in contrast to the pseudo hybridization of the ZNA probe. On the other hand, a 3.4 fold increase was recorded after the hybridization of the ZNA probe with the wDNA target. After the hybridization of the DNA probe and mDNA target (Figure 5), there was a 1.8 fold increase at R_ct_ obtained in contrast to the pseudo hybridization of the DNA probe. Similarly, a 1.8 fold increase at R_ct_ was observed in the presence of hybridization of the DNA probe with the wDNA target. According to the literature [50], the hybridization efficiency (HE%) provides information about the hybridization effectiveness. As a result, the hybridization efficiency (HE%) of our assay was also calculated and shown in Table S3. According to the HE% values, it was concluded that the ZNA probe exhibited a selective behavior to its complementary target where the DNA probe did not.

**Figure 6:**
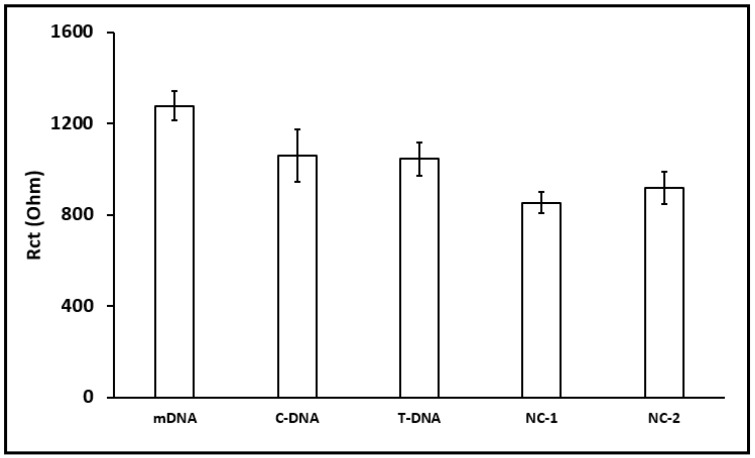
Histograms representing the average R_ct_ values related to the hybridization of ZNA probe with mDNA target, C-DNA, T-DNA, NC-1 or NC-2 (*n* = 3).

Alternating the incubations of the electrode with complementary and non-complementary DNA strands proved the specificity of the biorecognition interface. After hybridization of the ZNA/DNA probe with its complementary strand, an increase at R_ct_ is expected in comparison to the one measured for pseudo hybridization. On the other hand, the probe incubation with a non-complementary strand should yield with a negligible effect on the value of R_ct_. Similarly, a change at R_ct_ is expected in the case of the mismatch strand, however, not as much as the one obtained in the full match nucleic acid hybridization between the probe and its complements. Under this aim, the selectivity of the impedimetric array platform was tested and as a result, the average R_ct_ and HE% values were obtained after hybridization of the ZNA probe with the mDNA target/C-DNA/T-DNA/NC-1/NC-2. The results are given in Figure 6 and Table S4.

**Figure 7:**
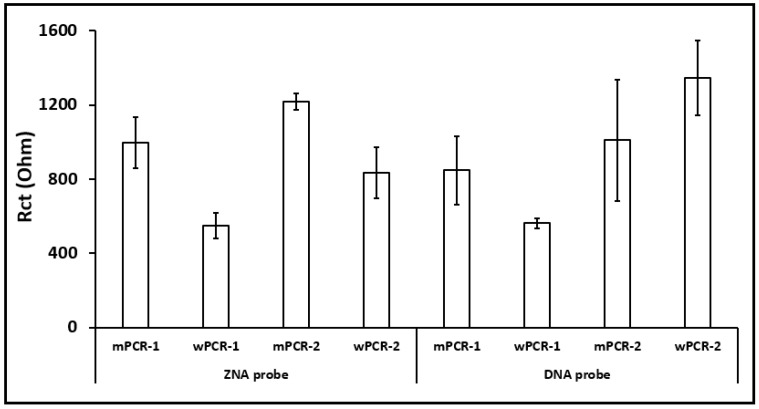
Histograms representing the average R_ct_ values related to the hybridization of the ZNA probe or DNA probe with mPCR-1, mPCR-2, wPCR-1 or wPCR-2 (*n* = 3).

The impedimetric detection of FV Leiden mutation in PCR products with the lengths of 143 nt and 220 nt were investigated. The results are given in Figure 7 and Table S3. The higher HE% value is expected in the case of hybridization of the ZNA probe with mPCR in comparison to the one with wPCR. In addition, the higher HE% is expected in the case of hybridization of the ZNA probe with its target DNA in contradiction of the DNA probe.

According to the HE% values (Table S5): (i) The ZNA probe selectively detected the single-base mutation, mPCR-1 (HE% = 80%) in comparison to wPCR-1 (HE% = 64%); (ii) a lower hybridization efficiency was calculated in the presence of the DNA probe in contrast to the ZNA probe; (iii) after the hybridization of the ZNA probe with mPCR-2, or wPCR-2, the HE% values were calculated and found to be 84% and 76%, respectively. However, the HE% values were found to be 53% and 65%, respectively after hybridization of the DNA probe with mPCR-2, or wPCR-2. In relation to hybridization effectiveness, this study concluded that the DNA probe could not present any selective behavior in the solution-phase hybridization process with mPCR-2/wPCR-2. Contrary to the DNA probe, the ZNA probe more selectively detected the single-base mutated target in mPCR-2 in comparison to wPCR-2.

## 4. Conclusions

While introducing a selective and sensitive assay based on the impedimetric detection of solution-phase nucleic acid hybridization related to FV Leiden mutation, the disposable electrodes enriched with carbon nanofibers (CNF-SPEs) were used for the first time in the present study. The experiments on solution-phase hybridization in the presence of SNPs were performed using both the ZNA and DNA probe, and consequently, a more stable and selective hybridization was achieved more effectively by using ZNA in contrast to the DNA probe.

These results are the proof of concept that the ZNA probe shows evidence of a quite selective behavior to SNPs by the advantage of replicating cationic spermine units that are increasing the affinity of ZNA probe to its target, while decreasing the electrostatic repulsion between negatively charged single stranded nucleic acids.

In the present study, the impedimetric detection of the FV Leiden mutation was performed in contribution of the ZNA probe which resulted in a relatively shorter time (i.e., 30 min) and selectively in comparison to earlier studies in the literature without using any type of labeled probe [23,24,25,36,37,38,50,51,52,53,54,55,56,57]. Some of these studies related to the electrochemical detection of FV Leiden mutation was presented in Table S6. The impedimetric detection of different types of single point mutation of G > A; G > C and G > T in short DNA oligonucleotides was successfully carried out herein. Moreover, the discrimination between mDNA (G to A) and wDNA was explored successfully with a high HE% even though the target sequence with a mutation (G to A) was at the 3‘-end position of both PCR products in the length of 143 nt or 220 nt. Besides, the impedimetric detection of any other SNPs (G to C or G to T) was performed efficiently by using the ZNA probe-based solution phase hybridization in contrast to the DNA probe.

The LOD was calculated and found to be 0.69 µg/mL (1.93 pmol in 20 µL sample). In addition to the advantages of impedimetric biosensors in terms of their cost per measurement, easy implementation, such as miniaturization and automation with on-site remote sensing capability, the lower LOD was achieved in the present study contrary to the ones obtained by optical methods [23], spectroscopic techniques [54] and electrochemical methods [36,37,38]. The ZNA probe based impedimetric assay in combination with CNF-SPEs can further expand the new generation nucleic acids for the development of point-of care (PoC) devices.

## Figures and Tables

**Table 1:** The anodic current I_a_ (µA), the cathodic current I_c_ (µA) of K_4_[Fe(CN)_6_]/K_3_[Fe(CN)_6_] measured by SPE and CNF-SPE with the surface area (A, cm^2^) of SPE and CNF-SPE.

		I_a_ (µA)	I_c_ (µA)	A (cm^2^)
	
**SPE**		39.70 ± 9.02	29.05 ± 3.37	0.118
**CNF-SPE**		43.8 ± 6.17	48.66 ± 2.37	0.132

## Supplementary Materials

The following are available online at https://www.mdpi.com/1996-1944/12/20/3377/s1. Figure S1: Nyquist diagrams obtained by (a) CNF-SPE, after pseudo hybridization of 2 µg/mL (b) 3’ZNA probe, (c) 5’ZNA probe, (d) DNA probe, (e) spermine, after hybridization of (f) 3’ZNA probe (g) 5’ZNA probe, (h) DNA probe, (i) spermine with 10 µg/mL mDNA target. Inset was the equivalent circuit model used for fitting of the impedance data. Figure S2: Nyquist diagrams obtained by (a) CNF-SPE, (b) pseudo hybridization of ZNA probe at 25 °C, (c) hybridization between 2 µg/mL ZNA probe and 10 µg/mL mDNA target at 25 °C, (d) pseudo hybridization of 5’ZNA probe at 50 °C, (e) hybridization between 2 µg/mL ZNA probe and 10 µg/mL mDNA target at 50 °C. Inset was the equivalent circuit model used for fitting of the impedance data. Figure S3: The Nyquist diagrams obtained after the hybridization of 2 µg/mL ZNA probe and 10 µg/mL mDNA target in PBS (pH 7.4), or PBS containing 0.5 mM Mg^2+^ (pH 7.40). (a) CNF-SPE, (b) the pseudo hybridization of ZNA probe in PBS, (c) the hybridization of ZNA probe and mDNA target in PBS, (d) the pseudo hybridization of 5’ZNA probe in PBS containing 0.5 mM Mg^2+^ (e) the hybridization of ZNA probe and mDNA target in PBS containing 0.5 mM Mg^2+^. Inset was the equivalent circuit model used for fitting of the impedance data. Figure S4: Nyquist diagrams obtained by (a) CNF-SPE, (b) the pseudo hybridization of ZNA probe in ABS, (c) the hybridization of ZNA probe and mDNA target in ABS, (d) the pseudo hybridization of 5’ZNA probe in PBS, (e) the hybridization of ZNA probe and mDNA target in PBS, (f) the pseudo hybridization of ZNA probe in CBS, (g) the hybridization of ZNA probe and mDNA target in CBS. Inset was the equivalent circuit model used for fitting of the impedance data. Figure S5: The Nyquist diagrams obtained by (a) CNF-SPE, (b) the pseudo hybridization of ZNA probe during 5 min, (c) the hybridization of ZNA probe and mDNA target during 5 min, (d) the pseudo hybridization of ZNA probe during 10 min, (e) the hybridization of ZNA probe and mDNA target during 10 min, (f) the pseudo hybridization of ZNA probe during 15 min, (g) the hybridization of ZNA probe and mDNA target during 15 min. Inset was the equivalent circuit model used for fitting of the impedance data. Figure S6: Nyquist diagrams of (a) CNF-SPE, after the hybridization of (b) 0.5 µg/mL, (c) 1 µg/mL (d) 2 µg/mL (e) 4 µg/mL ZNA prob and 10 µg/mL mDNA target. Inset was the equivalent circuit model used for fitting of the impedance data. Figure S7: Line graph representing the R_ct_ values recorded by the hybridization of 1 µg/mL ZNA probe with mDNA target at the concentration level from 0 to 14 µg/mL. Table S1: The R_ct_ values measured in the presence of the hybridization occurred between 1 µg/mL ZNA probe and 10 µg/mL mDNA target by single-use CNF-SPEs for three different days with the values of the average R_ct_ and the standard deviation with the RSD% for presenting the intra-day reproducibility. Table S2: The R_ct_ values measured in the presence of the hybridization occurred between 1 µg/mL ZNA probe and 10 µg/mL mDNA target by single-use CNF-SPEs for three different days with the values of the average R_ct_ and the standard deviation with the RSD% for presenting the inter-day reproducibility. Table S3: The hybridization efficiency (HE%) calculated based on the average R_ct_ value obtained after the hybridization of ZNA probe (or DNA probe) with mDNA target/wDNA target in contrast to the average R_ct_ value obtained in the presence of pseudo hybridization. Table S4: The hybridization efficiency (HE%) calculated based on the average R_ct_ value measured after the hybridization of ZNA probe with mDNA target/C-DNA/T-DNA/NC-1/NC-2 in contrast to the average R_ct_ value obtained in the presence of pseudo hybridization. Table S5: The hybridization efficiency (HE%) calculated based on the average R_ct_ value obtained after the hybridization of ZNA probe (or DNA probe) with mPCR-1/mPCR-2/wPCR-1/wPCR-2 in contrast to the average R_ct_ value obtained in the presence of pseudo hybridization. Table S6: The earlier studies developed for detection of Factor V Leiden mutation in contrast to the present study.
