# Supplementary material for: Electrochemical Detection of Solution Phase Hybridization Related to Single Nucleotide Mutation by Carbon Nanofibers Enriched Electrodes
Source: Materials (Basel). 2019 Oct 16;12(20):3377. doi: 10.3390/ma12203377 (PMC6829215; doi:10.3390/ma12203377)
Supplement: Supplementary file 1 [file materials-12-03377-s001.pdf]

materials

Supplementary Materials

Electrochemical Detection of Solution Phase Hybridization Related to Single Nucleotide Mutation by Carbon Nanofibers Enriched Electrodes

Arzum Erdem [1,2,*] and Ece Eksin [1,2]

[1] Faculty of Pharmacy, Analytical Chemistry Department, Ege University, Bornova, Izmir 35100, Turkey; eceksin@hotmail.com
[2] Biotechnology Department, Graduate School of Natural and Applied Sciences, Ege University, Bornova, Izmir 35100, Turkey
* Correspondence: arzum.erdem@ege.edu.tr

The base sequences of oligonucleotides and PCR products are listed below as given in our earlier reports [30–32];

5′. ZNA probe (Inosine substituted, 23 base):

5′-5S-AAT ACC TIT ATT CCT TIC CTI TC-3′ (S: Spermine, I: Inosine)

3′. ZNA probe (Inosine substituted, 23 base):

5′- AAT ACC TIT ATT CCT TIC CTI TC-5S-3′ (S: Spermine, I: Inosine)

DNA probe (Inosine substituted, 23 base):

5′-AAT ACC TIT ATT CCT TIC CTI TC-3′ (I: Inosine)

Complementary mutant type DNA target (mDNA, 23 base):

5′-GAC AGG C**A**A GGA ATA CAG GTA TT-3′

Wild type DNA oligonucleotide (wDNA, 23 base):

5′-GAC AGG C**G**A GGA ATA CAG GTA TT-3′

C-mDNA oligonucleotide (C-DNA, 23 base):

5′-GAC AGG C**C**A GGA ATA CAG GTA TT-3′

T-mDNA oligonucleotide (T-DNA, 23 base):

5′-GAC AGG C**T**A GGA ATA CAG GTA TT-3′

Noncomplementary DNA oligonucleotide-1 (NC-1, 20 base):

5′-AAT ACC ACA TCA TCC ATA TA-3′

Noncomplementary DNA oligonucleotide-2 (NC-2, 23 base):

5′-AAT ACC TGT ATT CCT CGC CTG TC-3′

Complementary mutant type PCR-1 (143 base, mPCR-1):

5′-ACC CAC AGA AAA TGA TGC CCA GTG CTT AAC AAG ACC ATA CTA CAG TGA CGT GGA CAT CAT GAG AGA CAT CGC CTC TGG GCT AAT AGG ACT ACT TCT AAT CTG TAA GAG CAG ATC CCT GGA CAG GCA AGG AAT ACA GGT ATT TT-3′

Wild type PCR-1 (143 base, wPCR-1):

5′-ACC CAC AGA AAA TGA TGC CCA GTG CTT AAC AAG ACC ATA CTA CAG TGA CGT GGA CAT CAT GAG AGA CAT CGC CTC TGG GCT AAT AGG ACT ACT TCT AAT CTG TAA GAG CAG ATC CCT GGA CAG GCG AGG AAT ACA GGT ATT TT-3′

Complementary mutant type PCR-2 (220 base, mPCR-2):

5′-ACC CAC AGA AAA TGA TGC CCA GTG CTT AAC AAG ACC ATA CT A CAG TGA CGT GGA CAT CAT GAG AGA CAT CGC CTC TGG GCT AAT AGG ACT ACT TCT AAT CTG TAA GAG CAG ATC CCT G A C AG TGA CGT GGA CAT CAT GAG AGA CAT CGC CTC TGG GCT AAT AGG ACT ACT TCT AAT CTG TAA GAG CAG ATC CCT GGA CAG GCA AGG AAT ACA GGT ATT TT-3′

Wild type PCR-2 (220 base, wPCR-2):

5′-ACC CAC AGA AAA TGA TGC CCA GTG CTT AAC AAG ACC ATA CT A CAG TGA CGT GGA CAT CAT GAG AGA CAT CGC CTC TGG GCT AAT AGG ACT ACT TCT AAT CTG TAA GAG CAG ATC CCT G A C AG TGA CGT GGA CAT CAT GAG AGA CAT CGC CTC TGG GCT AAT AGG ACT ACT TCT AAT CTG TAA GAG CAG ATC CCT GGA CAG GCG AGG AAT ACA GGT ATT TT-3′

ZNA probe stock solution as 472 μg/mL was prepared in Dulbecco's modified Phosphate Buffer Solution (pH 7.40) and kept frozen. The stock solutions of other oligonucleotides were prepared in ultrapure water (i.e, RNase/DNase free). The diluted solutions of ZNA probe, DNA probe, wDNA, mDNA, C-mDNA, T-mDNA, NC-1, NC-2 and PCR products were prepared in 50 mM phosphate buffer solution containing 20 mM NaCl (PBS, pH 7.40). The diluted solutions of mDNA target was prepared in PBS (pH 7.40), acetate buffer solution (ABS, pH 4.80), or carbonate buffer (CBS, pH 9.50), that was preferentially used according to the protocol followed herein. Other chemicals were in analytical reagent grade and they were supplied from Sigma-Aldrich and Merck.

Carbon nanofibers enriched screen printed electrodes (CNF-SPEs)

Graphitized carbon nanofibers enriched screen printed electrodes (CNF-SPEs) were purchased from DropSens (Oviedo-Asturias, Spain). A specific connector for CNF-SPEs (ref. DSC) purchased from DropSens (Oviedo-Asturias, Spain) that allows the connection of electrodes to the potentiostat.

After placing a 35 μL drop of the corresponding solution to the working area of these electrodes, each measurement was performed using CNF-SPEs similiarly introduced in our earlier reports [36–38].

The optimization of experimental parameters for impedimetric detection of FV Leiden mutation by CNF-SPEs:

The comparison of 5′ZNA probe, 3′ZNA probe and DNA probe

The selectivity of ZNA probe was firstly investigated. For this purpose, the impedimetric detection of mDNA target in the full match ZNA:DNA hybridization was performed in the presence of 3′ZNA probe, 5′ZNA probe, DNA probe, or spermine alone (Figure S1). After the pseudo-hybridization of 3′ ZNA probe, 5′ZNA probe, DNA probe the R_{ct} values were recorded as 290 Ohm, 228 Ohm, 1213 Ohm and 2.1 μA, respectively. On the other hand, after the hybridization of 3′ZNA probe, 5′ZNA probe and DNA probe with mDNA target, the R_{ct} values were recorded as 1215 Ohm, 1038 Ohm and 1564 Ohm, respectively. After the interaction of spermine and mDNA target, the R_{ct} value was 92% higher than the one obtained by the spermine in the absence of mDNA

target. The highest R_{ct} value was obtained in the presence of full match hybridization of 5′ZNA probe with mDNA target of all (shown in Figure S1-h), and the R_{ct} value was measured 355% higher than the ones recorded after the pseudo hybridization of 5′ZNA probe. It was concluded that the 5′ZNA probe presented more selective behavior than the one of 3′ZNA probe and DNA probe.

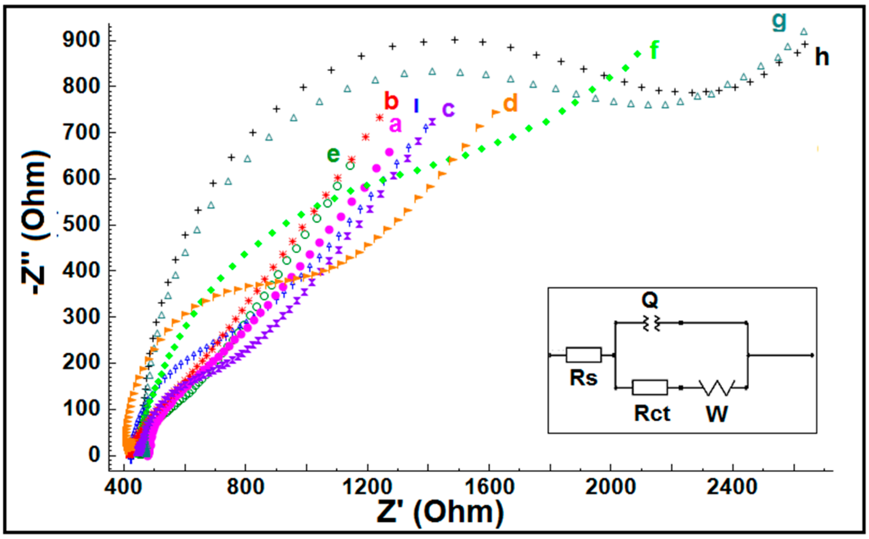

Figure S1. Nyquist diagrams obtained by **(a)** CNF-SPE, after pseudo hybridization of 2 µg/mL **(b)** 3′ZNA probe, **(c)** 5′ZNA probe, **(d)** DNA probe, **(e)** spermine, after hybridization of **(f)** 3′ZNA probe **(g)** 5′ZNA probe, **(h)** DNA probe, **(i)** spermine with 10 µg/mL mDNA target. Inset was the equivalent circuit model used for fitting of the impedance data.

The effect of temperature at hybridization process occurred between ZNA probe and mDNA target

The hybridization of 2 µg/mL ZNA probe and 10 µg/mL mDNA target was performed at 25 °C, or 50 °C during 10 min in PBS (pH 7.40) (Figure S2). Pseudo-hybridization of ZNA probe at 25 °C or 50 °C was also performed the R_{ct} value was measured as 246.50 Ohm and 344 Ohm. After the hybridization of 5′ZNA probe and mDNA target at 25 °C or 50 °C, the R_{ct} value was recorded as 1025.50 ± 17.68 Ohm (RSD%, 1%, n=2) and 1383.50 ± 89.80 Ohm (RSD%, 6%, n=2) respectively. The higher increase (as 316%) at R_{ct} value in contrast to the one obtained by pseudo hybridization was obtained in the case of the hybridization occurred at 25 °C. Thus, 25 °C was chosen as optimum temperature for hybridization.

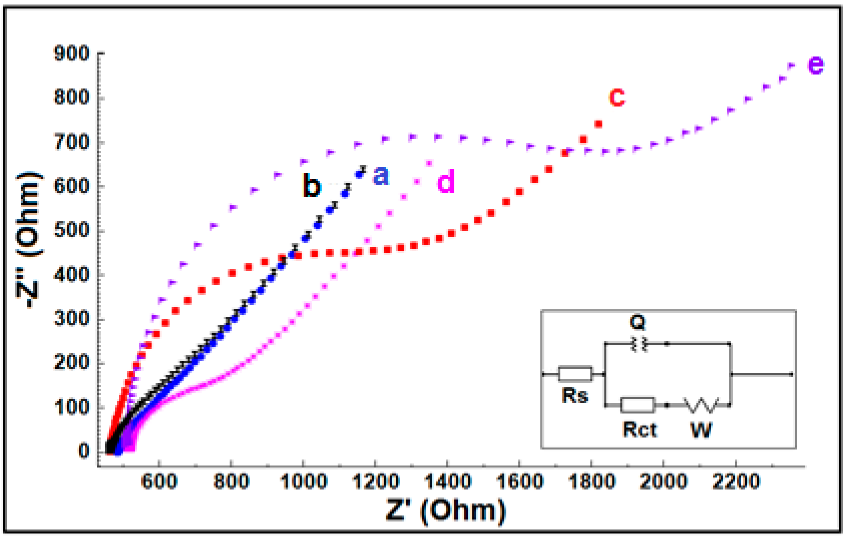

Figure S2. Nyquist diagrams obtained by **(a)** CNF-SPE, **(b)** pseudo hybridization of ZNA probe at 25 °C, **(c)** hybridization between 2 μg/mL ZNA probe and 10 μg/mL mDNA target at 25 °C, **(d)** pseudo hybridization of 5′ZNA probe at 50 °C, **(e)** hybridization between 2 μg/mL ZNA probe and 10 μg/mL mDNA target at 50 °C. Inset was the equivalent circuit model used for fitting of the impedance data.

The effect of Mg^{+2} concentration at hybridization process

The effect of $MgCl_2$ concentration upon the hybridization of ZNA probe with mDNA target was studied (Figure S3). For this purpose, the hybridization was performed during 10 min in the solution of PBS (pH 7.40), or PBS containing 0.5 mM Mg^{+2}. As seen in Figure S3, , the highest R_{ct} value was measured as 1025.50 ± 17.68 Ohm (RSD%, 1%, n=2) in PBS (pH 7.40) without Mg^{+2} .

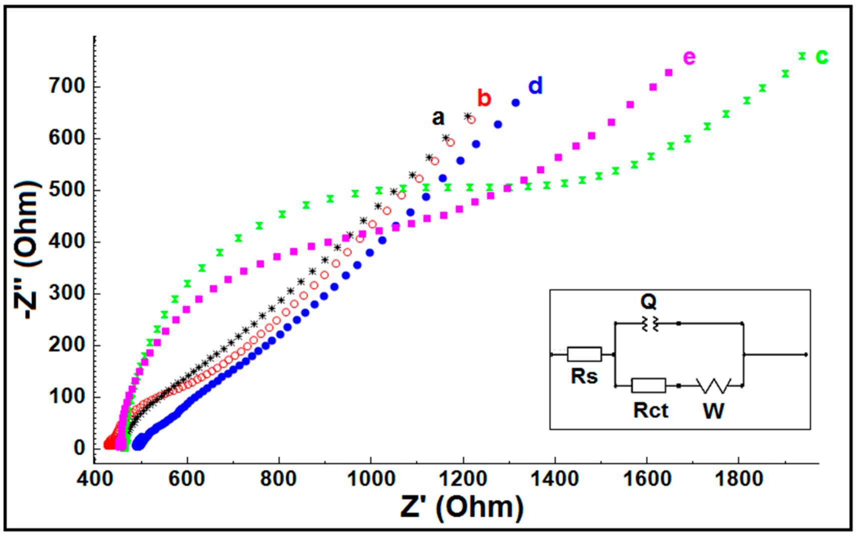

Figure S3. The Nyquist diagrams obtained after the hybridization of 2 μg/mL ZNA probe and 10 μg/mL mDNA target in PBS (pH 7.4), or PBS containing 0.5 mM Mg^{2+} (pH 7.40). **(a)** CNF-SPE, **(b)** the pseudo hybridization of ZNA probe in PBS, **(c)** the hybridization of ZNA probe and mDNA target in PBS, **(d)** the pseudo hybridization of 5′ZNA probe in PBS containing 0.5 mM Mg^{2+} **(e)** the hybridization of ZNA probe and mDNA target in PBS containing 0.5 mM Mg^{2+}. Inset was the equivalent circuit model used for fitting of the impedance data.

The effect of pH at hybridization process

The effect of pH upon the hybridization process was evaluated and the results were shown in Figure S4. The hybridization between 2 μg/mL ZNA probe and 10 μg/mL (equals to 1.4 μM) mDNA target was done in ABS (pH 4.80), PBS (pH 7.40), or CBS (pH 9.50). The pseudo-hybridization of ZNA probe in ABS (pH 4.80), PBS (pH 7.40), or CBS (pH 9.50) was also studied and the R_{ct} values were measured as 271 Ohm, 248 ± 18.68 Ohm (RSD%, 7.53%, n=3) and 440 Ohm, respectively (shown in Figure S4). After hybridization in ABS (pH 4.80), PBS (pH 7.40), or CBS (pH 9.50), the R_{ct} values were measured as 702 Ohm, 1132 ± 184.89 Ohm (RSD%, 16.33%, n=3) and 473 Ohm, respectively. It was concluded that the hybridization could be occurred efficiently in the medium of PBS with pH 7.40 since the highest increase (356%) at the R_{ct} was obtained in hybridization of ZNA:DNA performed in PBS medium.

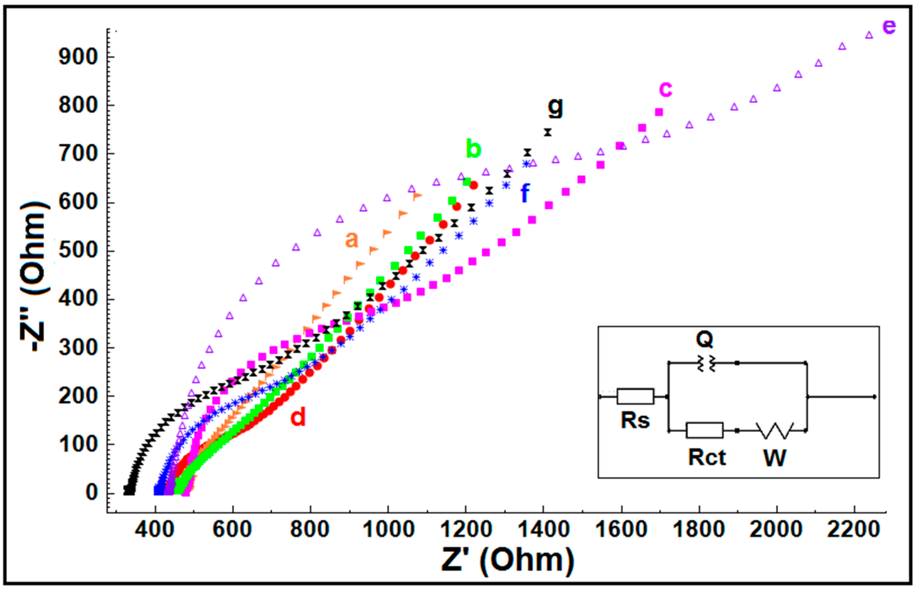

Figure S4. Nyquist diagrams obtained by (a) CNF-SPE, (b) the pseudo hybridization of ZNA probe in ABS, (c) the hybridization of ZNA probe and mDNA target in ABS, (d) the pseudo hybridization of 5′ZNA probe in PBS, (e) the hybridization of ZNA probe and mDNA target in PBS, (f) the pseudo hybridization of ZNA probe in CBS, (g) the hybridization of ZNA probe and mDNA target in CBS. Inset was the equivalent circuit model used for fitting of the impedance data.

The effect of hybridization time at hybridization process

The hybridization between ZNA probe and mDNA target was performed during 5, 10 and 15 min. The highest increase at the R_{ct} value was calculated as 356% according to the changes at R_{ct} after hybridization of ZNA:DNA in 10 min (Figure S5). Thus, 10 min was used as the optimum hybridization time in further studies.

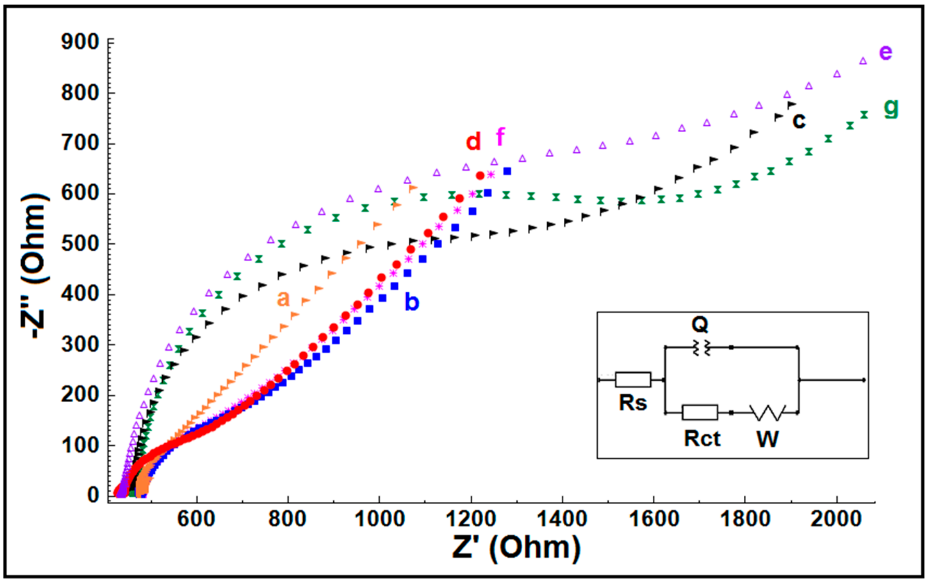

Figure S5. The Nyquist diagrams obtained by **(a)** CNF-SPE, **(b)** the pseudo hybridization of ZNA probe during 5 min, **(c)** the hybridization of ZNA probe and mDNA target during 5 min, **(d)** the pseudo hybridization of ZNA probe during 10 min, **(e)** the hybridization of ZNA probe and mDNA target during 10 min, **(f)** the pseudo hybridization of ZNA probe during 15 min, **(g)** the hybridization of ZNA probe and mDNA target during 15 min. Inset was the equivalent circuit model used for fitting of the impedance data.

The effect of ZNA probe concentration at hybridization process:

In order to find optimum concentration of ZNA probe, the hybridization was performed in various concentrations of ZNA probe (0.5, 1, 2 and 4 µg/mL) and 10 µg/mL (equals to 1.4 µM) mDNA target and accordingly, the R_{ct} value was measured (shown in Figure S6). The highest R_{ct} value was recorded as 1355 ± 4.24 Ohm (RSD%, 4.24%, n=2) in the presence of 1 µg/mL ZNA probe. Therefore, 1 µg/mL was chosen as optimum ZNA probe concentration for the further studies.

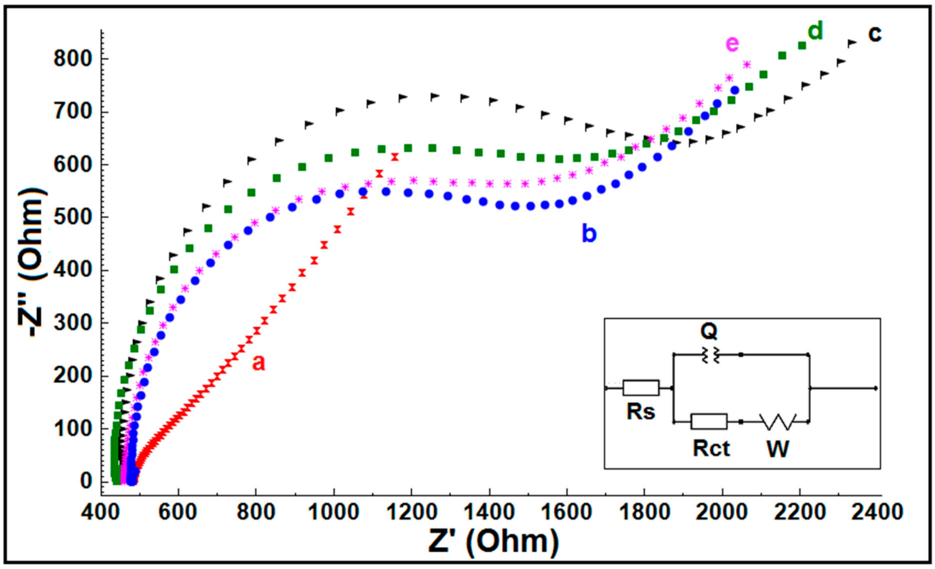

Figure S6. Nyquist diagrams of **(a)** CNF-SPE, after the hybridization of **(b)** 0.5 µg/mL, **(c)** 1 µg/mL **(d)** 2 µg/mL **(e)** 4 µg/mL ZNA prob and 10 µg/mL mDNA target. Inset was the equivalent circuit model used for fitting of the impedance data.

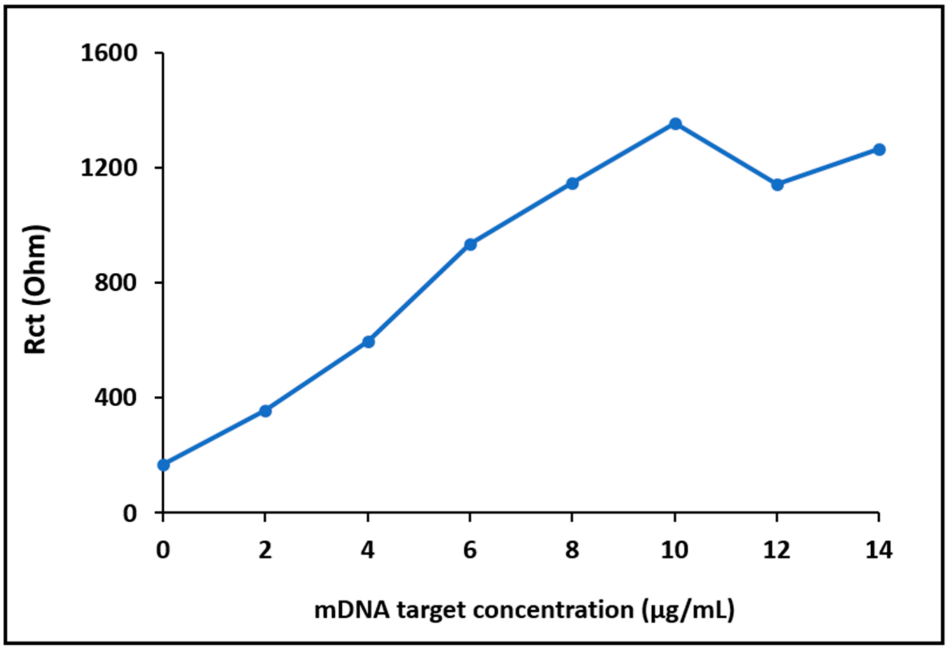

Figure S7. Line graph representing the R_{ct} values recorded by the hybridization of 1 µg/mL ZNA probe with mDNA target at the concentration level from 0 to 14 µg/mL.

The hybridization efficiency (HE%) was calculated for each hybridization occurred between DNA probe/ZNA probe with mDNA/wDNA, mPCR-1/wPCR-1, or mPCR-2/mPCR-2 according to the equation 1.

$$\text{The hybridization efficiency (HE\%)} = [\Delta R_{ct} / R_{ct\ hybrid}] \times 100 \quad (\Delta R_{ct} = R_{ct\ hybrid} - R_{ct\ probe}) \tag{1}$$

The hybridization efficiency (HE%) was calculated based on the results obtained by CNF-SPE related to each experiment on possible hybridization between DNA probe/ZNA probe with mDNA/wDNA target or mPCR/wPCR (Equation 1). The higher HE% value is expected in the case of hybridization of ZNA probe with mDNA/mPCR comparison to the one with wDNA/wPCR. In addition, the higher HE% is expected in case of hybridization between ZNA probe and its target DNA in contrast to DNA probe. Therefore, we can consider that ZNA probe can recognize SNP in NA hybridization more selectively than DNA probe.

Table S1. The R_{ct} values measured in the presence of the hybridization occurred between 1 μg/mL ZNA probe and 10 μg/mL mDNA target by single-use CNF-SPEs for three different days with the values of the average R_{ct} and the standard deviation with the RSD% for presenting the intra-day reproducibility.

	**intra-day reproducibility**		
	**1st day**	**2nd day**	**3rd day**
	1358	1266	1352
	1250	1212	1229
**Average (Ohm)**	**1304.00**	**1239.00**	**1290.50**
**Standard deviation**	**76.37**	**38.18**	**86.97**
**RSD%**	**5.86**	**3.08**	**6.74**

Table S2. The R_{ct} values measured in the presence of the hybridization occurred between 1 μg/mL ZNA probe and 10 μg/mL mDNA target by single-use CNF-SPEs for three different days with the values of the average R_{ct} and the standard deviation with the RSD% for presenting the inter-day reproducibility.

	**inter-day reproducibility**	
	1358	**1st day**
	1250	
	1266	**2nd day**
	1212	
	1352	**3rd day**
	1229	
**Average (Ohm)**	**1277.83**	
**Stansard deviation**	**62.55**	
**RSD%**	**4.89**	

Table S3. The hybridization efficiency (HE%) calculated based on the average R_{ct} value obtained after the hybridization of ZNA probe (or DNA probe) with mDNA target / wDNA target in contrast to the average R_{ct} value obtained in the presence of pseudo hybridization.

	**R_{ct} (Ohm)**	**HE%**
**ZNA probe**	199 ± 52.07	-
**ZNA probe - mDNA Target**	1277.83 ± 62.55	84
**ZNA probe - wDNA Target**	947.67 ± 195.81	79
**DNA probe**	472 ± 46.67	-
**DNA probe - mDNA Target**	1333.50 ± 31.82	65
**DNA probe - wDNA Target**	1325 ± 9.90	64

Table S4. The hybridization efficiency (HE%) calculated based on the average R_{ct} value measured after the hybridization of ZNA probe with mDNA target/ C-DNA/ T-DNA /NC-1 / NC-2 in contrast to the average R_{ct} value obtained in the presence of pseudo hybridization.

	**R_{ct} (Ohm)**	**HE%**
**ZNA probe**	199 ± 52.07	-
**ZNA probe - mDNA Target**	1277.83 ± 62.55	84
**ZNA probe - C-DNA**	1059 ± 113.14	81
**ZNA probe - T-DNA**	1044.5 ± 71.42	80
**ZNA probe - NC-1**	852 ± 46.67	76
**ZNA probe - NC-2**	917 ± 70.71	78

Table S5. The hybridization efficiency (HE%) calculated based on the average R_{ct} value obtained after the hybridization of ZNA probe (or DNA probe) with mPCR-1/ mPCR-2 / wPCR-1/ wPCR-2 in contrast to the average R_{ct} value obtained in the presence of pseudo hybridization.

	**R_{ct} (Ohm)**	**HE%**
**ZNA probe**	199 ± 52.07	-
**ZNA probe – mPCR-1**	995 ± 137.1	80
**ZNA probe – wPCR-1**	549.5 ± 70	64
**ZNA probe – mPCR-2**	1219.7 ± 45	84
**ZNA probe – wPCR-2**	835.5 ± 137.9	76
**DNA probe**	472 ± 46.67	-
**DNA probe – mPCR-1**	847 ± 185.2	44
**DNA probe – wPCR-1**	562 ± 26.8	16
**DNA probe – mPCR-2**	1011 ± 326.6	53
**DNA probe – wPCR-2**	1347.5 ± 201.5	65

Table S6. The earlier studies developed for detection of Factor V Leiden mutation in contrast to the present study.

**Method**	**Label**	**Assay time (min)**	**DL**	**Selectivity**	**Implementation to chip system**	**Ref**
Immuno-optical	HRP labeled IgG	10	NA	NA	No	[23]
Electrochemical	-	22	60.31 fM	tested to Wild type DNA	No	[24]
Electrochemical	AuNP capped capture probe / Inosine-substitut ed probe	180	0.83 fM	tested to Wild type DNA	No	[25]
Electrochemical	-	135	376 nM	tested to Wild type DNA as well as other DNA with point mutations (G>C, G>T), non complementary DNA	Yes	[36]
Electrochemical	-	60	207 nM	tested to Wild type DNA as well as other DNA with point mutations (G>C, G>T), non complementary DNA	Yes	[37]
Electrochemical	-	30	1.17 μM	tested to Wild type DNA as well as other DNA with point mutations	Yes	[38]

				(G>C, G>T), non complementary DNA		
PCR	-	127	NA	tested to non-mutated DNA, heterozygous and homozygous carriers	No	[45]
PCR-RFLP	Anthraquinone labeled LNA	60	NA	tested to Wild type DNA	No	[48]
Electrochemical	-	30	96.5 nM	tested to Wild type DNA as well as other DNA with point mutations (G>C, G>T), non complementary DNA	Yes	Present work
